# When the Stomach Takes a Vacation: The Unseen Battles of Gastroparesis

**DOI:** 10.7759/cureus.56263

**Published:** 2024-03-16

**Authors:** Beatriz R. Sousa, Teresa B Rodrigues, José Ribeiro

**Affiliations:** 1 Internal Medicine, Hospital de São José, Unidade Local de Saúde São José, Lisbon, PRT; 2 Radiology, Hospital da Luz, Lisbon, PRT

**Keywords:** motility disorders, adult internal medicine, geriatric age, quality of life (qol), gastroparesis

## Abstract

Gastroparesis is a syndrome characterised by delayed gastric emptying that is usually idiopathic, diabetic, or iatrogenic. This underdiagnosed disease has a substantial influence on the quality of life of its patients. We present the case of an 86-year-old man with dementia, benign prostatic hyperplasia, and gastroesophageal reflux disease who developed symptoms of gastroparesis during a lengthy hospital stay. Computed tomography (CT) and upper digestive endoscopy demonstrated gastric distention and pyloric stenosis. Despite cautious treatment and eventual pyloric dilation, the patient died from aspiration due to refractory respiratory failure. This example emphasises the need for early detection and thorough examination of gastroparesis to optimise patient outcomes and reduce morbidity and mortality.

## Introduction

Gastroparesis is a syndrome of objectively delayed gastric emptying, with the majority of cases being idiopathic, diabetic, or iatrogenic [1, 2]. This clinical entity is underdiagnosed. Epidemiological studies conducted in the USA and the UK have revealed a prevalence ranging from 267.7 to 338.7 and 13.8 per 100,000 persons, respectively [2, 3].

Diagnosing gastroparesis in the geriatric population can be challenging due to the frequent presentation of non-specific symptoms when compared with younger age groups [4]. The investigation for the etiological cause must include haemoglobin, fasting glucose, serum total protein, albumin, and thyrotropin concentrations, as well as esophagogastroduodenoscopy (EGD), computed tomography (CT), and scintigraphy [1, 5].

In most cases, the cause of gastroparesis is persistent, requiring ongoing symptomatic care and resulting in a significant patient burden, with a negative correlation between symptom severity and patient quality of life, particularly in geriatric patients [5, 6].

## Case presentation

An 86-year-old man with dementia, benign prostatic hyperplasia, and gastroesophageal reflux disease underwent prolonged hospitalisation attributable to social-related factors. Over the course of this extended stay, the patient developed symptoms of nausea, vomiting, early satiety, and abdominal bloating. A physical examination revealed significant distention and tenderness in the upper abdomen.

**Figure 1:**
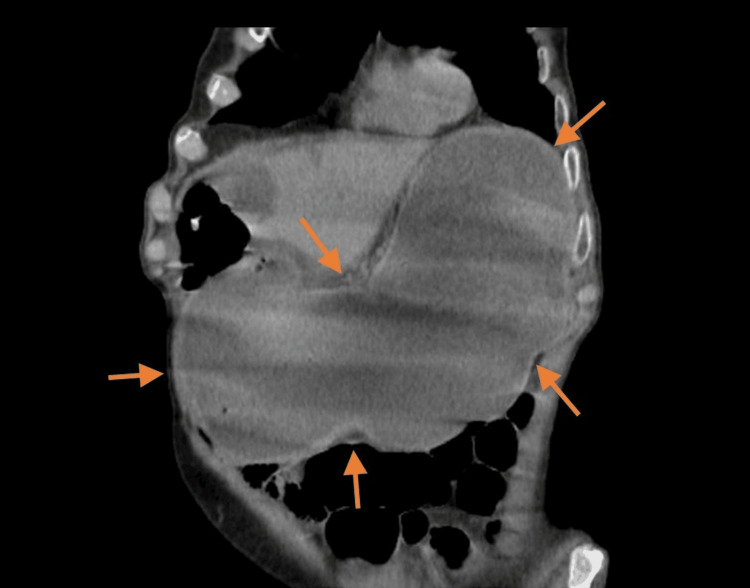
An abdominal CT scan shows gastric dilatation (arrows).

A high-volume stomach distention filled with fluid and food content was shown on CT (Figure 1), with an abnormal duodenal arch suggestive of a gastric outlet obstruction due to pyloric stenosis. Findings from the EGD indicated evidence of pyloric spasm, with a gastric biopsy that excluded neoplasia.

After a first attempt with a conservative approach, gastroscopy with pyloric dilatation and placement of a transpyloric tube was performed in response to persisting discomfort. During hospitalisation, the patient died due to aspiration with refractory respiratory failure.

## Discussion

This case highlights the challenges faced in the diagnosis and management of gastroparesis in elderly patients. Gastroparesis may present additional complications due to a variety of factors, including inadequate symptom communication due to underlying cognitive impairments. Non-specific signs and symptoms of gastroparesis in the elderly, such as nausea, vomiting, early satiety, and abdominal distension, can be attributed to a variety of age-related disorders, such as constipation, creating diagnostic confusion. These difficulties emphasise the importance of having a high level of suspicion and doing a comprehensive evaluation of older patients who present with gastrointestinal problems.

Managing gastroparesis in the elderly requires a specialised approach that takes into consideration the population's specific needs and vulnerabilities, with a multidisciplinary team [7]. Conservative interventions such as dietary changes and prokinetic drugs may be used, taking into account pharmaceutical interactions and frailty [8]. A multidisciplinary team can personalise therapy regimens to improve patient outcomes and quality of life.

## Conclusions

The authors have presented this case to highlight the underdiagnosis of gastroparesis and to emphasize the importance of identifying the cause and conducting an aetiological investigation. For prompt intervention, healthcare providers' awareness is essential. Due to the difficulties that older patients may have in communicating their symptoms, physicians’ assessments require closer attention to non-verbal communication. The difficulties this case brings emphasize how important it is for healthcare providers to continue being watchful when diagnosing gastroparesis in older patients, especially when there are coexisting diseases that could make diagnosis challenging. The only way to improve patients' quality of life and decrease mortality and morbidity is through a multidisciplinary approach.
